# Characterization of pathological changes in the olfactory system of mice exposed to methylmercury

**DOI:** 10.1007/s00204-024-03682-w

**Published:** 2024-02-17

**Authors:** Yuta Iijima, Ryohei Miki, Nobumasa Takasugi, Masatake Fujimura, Takashi Uehara

**Affiliations:** 1 Department of Medicinal Pharmacology, Graduate School of Medicine, Dentistry and Pharmaceutical Sciences, Okayama University, Okayama, 700‑8530 Japan; 2 Department of Basic Medical Science, National Institute for Minamata Disease, Kumamoto, 867‑0008 Japan

**Keywords:** Methylmercury, Minamata disease, Olfactory dysfunction, Neuronal cell death, Glial activation

## Abstract

Methylmercury (MeHg) is a well-known environmental neurotoxicant that causes severe brain disorders such as Minamata disease. Although some patients with Minamata disease develop olfactory dysfunction, the underlying pathomechanism is largely unknown. We examined the effects of MeHg on the olfactory system using a model of MeHg poisoning in which mice were administered 30 ppm MeHg in drinking water for 8 weeks. Mice exposed to MeHg displayed significant mercury accumulation in the olfactory pathway, including the nasal mucosa, olfactory bulb, and olfactory cortex. The olfactory epithelium was partially atrophied, and olfactory sensory neurons were diminished. The olfactory bulb exhibited an increase in apoptotic cells, hypertrophic astrocytes, and amoeboid microglia, mainly in the granular cell layer. Neuronal cell death was observed in the olfactory cortex, particularly in the ventral tenia tecta. Neuronal cell death was also remarkable in higher-order areas such as the orbitofrontal cortex. Correlation analysis showed that neuronal loss in the olfactory cortex was strongly correlated with the plasma mercury concentration. Our results indicate that MeHg is an olfactory toxicant that damages the central regions involved in odor perception. The model described herein is useful for analyzing the mechanisms and treatments of olfactory dysfunction in MeHg-intoxicated patients.

**Supplementary Information:**

The online version contains supplementary material available at 10.1007/s00204-024-03682-w.

## Introduction

Olfaction plays a pivotal role in daily life by influencing eating habits, helping to avoid environmental hazards, and enhancing social communication. However, olfaction is frequently disrupted by various factors (Schafer et al. 2021; Stevenson 2010). For example, the olfactory system is uniquely vulnerable to environmental neurotoxicants because olfactory sensory neurons (OSNs) are physically exposed to the external environment. Animal studies have demonstrated that certain pesticides, air pollutants, and heavy metals can damage the olfactory epithelium, resulting in olfactory dysfunction (Genc et al. 2012; Sunderman 2001; Werner and Nies 2018). Neurodegenerative diseases such as Alzheimer’s disease and Parkinson’s disease also cause olfactory dysfunction secondary to damage in brain areas involved in olfactory processing, such as the olfactory bulb and entorhinal cortex (ENT). These impairments appear before the primary symptoms, suggesting that olfaction is a clinical marker of neurodegenerative disease (Doty 2012; Murphy 2019). Thus, assessment of the olfactory system is crucial in evaluating the toxicity of environmental chemicals and may also be a valuable model for analyzing the pathomechanism of brain disorders.

Mercury and mercury compounds have been widely used in industrial and medical applications since ancient times. However, mercury is listed by the World Health Organization as one of the top ten chemicals of public health concern because of its toxicity and frequency of exposure. Most attention regarding mercury pollution is focused on methylmercury (MeHg). MeHg is produced from inorganic mercury mainly by sulfur-reducing bacteria in aquatic sediments (Compeau and Bartha 1985; King et al. 2000). Because of its high accumulation in the aquatic food chain through biomagnification, ingestion of fish and their predators is the main source of exposure to MeHg in humans (Hong et al. 2012). MeHg easily crosses the blood–brain barrier and exerts neurotoxicity that can lead to serious brain disorders such as Minamata disease (MD). Patients with MD have region-specific neuronal damage predominantly in the cerebellum and cerebrum, such as the postcentral gyrus (somatosensory area), precentral gyrus (motor area), calcarine sulcus (visual area), and superior temporal gyrus (auditory area) (Eto 1997; Eto and Takeuchi 1978). Consistent with these lesions, patients with MD develop characteristic signs and symptoms including ataxia, visual field constriction, and somatosensory and auditory disturbances (Harada 1995). Several studies have also shown that patients with MD exhibit olfactory dysfunction (Furuta et al. 1994). However, the causative lesions have not been thoroughly investigated, and how MeHg affects the olfactory system remains unclear.

We hypothesized that olfactory dysfunction in cases of MeHg poisoning mainly results from brain disorders in association with the following factors. (1) The risk of inhalation exposure to toxic doses of MeHg is low when ingesting MeHg-containing solutions. (2) MeHg is easily transferred to the brain and irreversibly damages the central nervous system. (3) MD causes central sensory disturbances that affect visual and auditory perception. (4) Certain neurodegenerative diseases cause olfactory dysfunction. In the present study, we used a well-established mouse model of MeHg poisoning to investigate the effects of MeHg on the olfactory system and elucidate the pathomechanism of olfactory dysfunction in MeHg poisoning.

## Materials and methods

### Animals

Male C57BL/6NJcl mice were purchased from CLEA Japan (Tokyo, Japan). All mice were housed in plastic cages (three animals per cage) and allowed free access to chow (CE-2; CLEA Japan) and water. The animal facility was kept at 25℃ ± 2 °C with a relative humidity of 65% ± 5% under a 12-h light/dark cycle. The mice were euthanized by cardiac blood sampling under deep anesthesia with isoflurane and transcardially perfused with saline.

### MeHg administration

Two-month-old male mice were randomly divided into two groups: a vehicle group and an MeHg-exposed group. MeHg (Tokyo Chemical Industry, Tokyo, Japan) was dissolved at 30 ppm in distilled water containing equimolar amounts of glutathione (Fujifilm Wako Pure Chemical, Osaka, Japan) and administered via drinking water for 8 weeks as previously described (Fujimura et al. 2009). The vehicle group received drinking water containing only glutathione.

### Measurement of mercury content

Whole blood was heparinized immediately after sampling and separated into plasma and other fractions by centrifugation. The nasal mucosa and right brain were excised, followed by resection of the olfactory bulb, olfactory tubercle (OT), and piriform cortex (PIR) from the right brain. Next, 19 μL of a 5 N NaOH solution was added per 1 mg tissue, incubated at 70 °C for 30 min to completely lyse the tissue, and neutralized with 5 N HCl. The mercury concentration was determined with the oxygen combustion–gold amalgamation method using an MA-2 mercury analyzer (Nippon Instruments, Tokyo, Japan) with MA2000 software version 1.7.8. (Nippon Instruments) as previously described (Hiraoka et al. 2021).

### Hindlimb extension test

Hindlimb impairment is a marker of disease progression in rodent models of MeHg poisoning (Fujimura and Usuki 2020; Nomura et al. 2022). To evaluate hindlimb impairment by MeHg poisoning, the mice were suspended by their tail and the extent of hindlimb extension was observed for 15 s. If both hindlimbs spread widely outward from the abdomen, a score of 0 was assigned (normal phenotype). If one hindlimb was retracted or both hindlimbs were partially retracted toward the abdomen without touching it, a score of − 1 was assigned (mild defect). If both hindlimbs were partially retracted toward the abdomen without touching each other, a score of − 2 was assigned (moderate defect). If both hindlimbs were fully clasped toward the abdomen and touching each other, a score of − 3 was assigned (severe defect).

### Preparation of tissue sections

The left brain tissue was fixed with 4% paraformaldehyde in 0.1 M phosphate buffer immediately after dissection. After the tissue had been embedded in paraffin, serial sagittal sections of 5-μm thickness were prepared using a microtome and mounted on glass slides. The heads of other mice were excised and fixed with 4% paraformaldehyde in 0.1 M phosphate buffer. Nasal tissues were harvested from the skull and decalcified with 20% ethylenediaminetetraacetic acid solution (pH 7.0) for 20 days. After the tissue had been embedded in paraffin, serial coronal sections of 5-μm thickness were prepared using a microtome and mounted on glass slides. The tissue sections were deparaffinized in xylene and rehydrated in a graded series of ethanol solutions before staining.

### Histological analysis

Staining with hematoxylin (Dako, Carpinteria, CA, USA) and eosin (Sakura Finetek Japan, Tokyo, Japan) was performed to analyze the olfactory epithelium structures. Coronal sections of the nasal cavity were divided into six subareas: the lateral turbinate (areas 1 and 2), medial turbinate (areas 1 and 2), and dorsal and ventral nasal septum. The thickness of the olfactory epithelium was measured between the surface of the epithelium and the line of basal cells in all bilateral subareas, and their mean values were calculated for each animal. Immunohistochemistry was performed using a VECTASTAIN Elite ABC Kit (Vector Laboratories, Newark, NJ, USA) according to the manufacturer’s instructions with the following primary antibodies: rabbit anti-glial fibrillary acidic protein (GFAP) (#60-0032-7, 1:2; Genemed Biotechnologies, South San Francisco, CA, USA), rabbit anti-ionized calcium-binding adapter molecule 1 (Iba1) (#013-27691, 1:250; Fujifilm Wako Pure Chemical), and rabbit anti-neuronal nuclei (NeuN) (#GTX133127, 1:250; GeneTex, Irvine, CA, USA). Deparaffinized sections were boiled for 20 min in 10 mM citrate buffer with a pH of 6 (Genemed Biotechnologies) for antigen retrieval, and endogenous peroxidase activity was blocked with 3% hydrogen peroxide in methanol for 20 min at room temperature before immunohistochemistry. After antigen–antibody reaction, the sections were developed using a 3,3′-diaminobenzidine Substrate Kit (Vector Laboratories) according to the manufacturer’s instructions. The sections were counterstained with hematoxylin for GFAP and Iba1 immunohistochemistry and mounted using Eukitt mounting media (ORSAtec, Bobingen, Germany). All images were captured and analyzed using a BX50 microscope (Evident, Tokyo, Japan) with FLOVEL image filling system version 2.30.03 (Flovel, Tokyo, Japan) for brain tissues and using a VS200 slide scanner (Evident) with OlyVIA image viewer software version 4.1 (Evident) for nasal tissues. The detailed brain regions were identified using a brain map of adult mice published online by the Allen Institute for Brain Science (http://atlas.brain-map.org/).

### TUNEL staining

Apoptosis-induced cell death was monitored by the terminal deoxynucleotidyl transferase-mediated dUTP nick-end labeling (TUNEL) assay using an In Situ Cell Death Detection Kit, TMR red (#12,156,792,910; Roche, Basel, Switzerland) according to the manufacturer’s instructions. Briefly, deparaffinized sections were permeabilized with 20 μg/mL Proteinase K (Qiagen, Venlo, Nederland) in 10 mM Tris–HCl (pH 7.5) for 20 min at 37 °C. After washing in phosphate-buffered saline (PBS), the sections were incubated with TUNEL reaction mixture for 1 h at 37 °C, washed in PBS, and mounted in VECTASHIELD Vibrance Antifade Mounting Medium with 4′,6-diamidino-2-phenylindole (DAPI) (Vector Laboratories). All images were captured and analyzed using the ECLIPSE Ti confocal microscope (Nikon Instruments, Tokyo, Japan) with NIS-Elements AR imaging software version 4.00.06 (Nikon Instruments). TUNEL-positive cells were identified by TMR red signals in the nucleus stained with DAPI.

### Immunofluorescence

Deparaffinized sections were boiled for 20 min in 10 mM citrate buffer (pH 6) (Genemed Biotechnologies) for antigen retrieval, rinsed in distilled water, and then blocked with 3% normal goat serum (Vector Laboratories) in PBS for 1 h at room temperature. The sections were incubated with primary antibodies in PBS or Can Get Signal immunostain solution (Toyobo, Osaka, Japan) for 1 h at room temperature. The primary antibodies were as follows: rabbit anti-GFAP, rabbit anti-Iba1, rabbit anti-NeuN, and rabbit anti-olfactory marker protein (OMP) (#ab183947, 1:1,000; Abcam, Cambridge, UK). After washing in PBS, the sections were incubated with Alexa Fluor 488- or 594-conjugated goat anti-rabbit IgG antibodies (#A31627 and #A-11012, 1:1,000; Thermo Fisher Scientific, Waltham, MA, USA) in PBS or Can Get Signal immunostain solution for 1 h at room temperature, washed in PBS, and mounted using VECTASHIELD Vibrance Antifade Mounting Medium with DAPI (Vector Laboratories). All images were captured and analyzed using the ECLIPSE Ti confocal microscope (Nikon Instruments) with NIS-Elements AR imaging software version 4.00.06 (Nikon Instruments). The number of OMP-positive cells was measured in all bilateral subareas, and their mean values were calculated for each animal.

### Statistical analysis

Quantitative data are presented as the means ± standard error of the mean (s.e.m.). Statistical analyses were performed using GraphPad Prism software version 10.0.2 (GraphPad Software, San Diego, CA, USA). Differences between two means were analyzed using a two-tailed Student’s *t*-test and two-way analysis of variance (ANOVA) followed by Bonferroni’s post hoc test. Pearson’s correlation analysis was performed to evaluate the correlation between the number of NeuN-positive cells in the region of interest and mercury concentration in plasma. All *p*-values of < 0.05 were considered statistically significant.

## Results

### Accumulation of mercury in the olfactory pathway after MeHg exposure

**Fig. 1:**
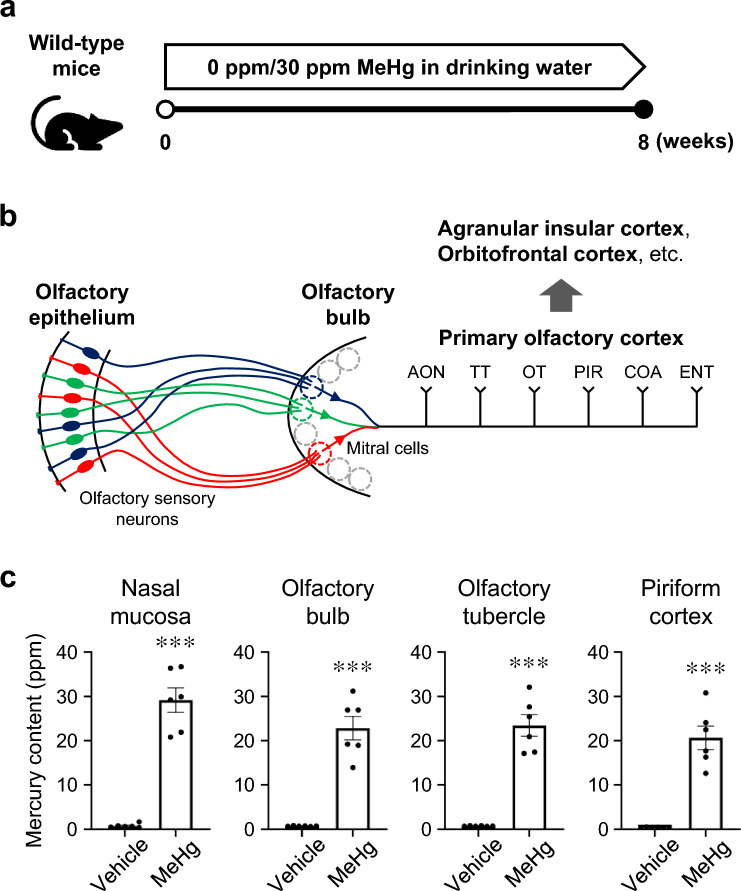
Accumulation of mercury in the olfactory pathway. **a** Schematic of subchronic exposure to methylmercury (MeHg) in wild-type mice. Either 0 ppm (vehicle) or 30 ppm MeHg was administered in the drinking water. After 8 weeks of MeHg exposure, the mice were sacrificed for further analysis. **b** Schematic of the main olfactory system. Each olfactory sensory neuron expresses only one type of olfactory receptor. Neurons expressing the same receptor project to specific glomeruli within the olfactory bulb and form synapses with mitral cells and tufted cells (not shown). The axons of mitral/tufted cells project to the primary olfactory cortex, including the anterior olfactory nucleus (AON), tenia tecta (TT), olfactory tubercle (OT), piriform cortex (PIR), cortical amygdala (COA), and entorhinal cortex (ENT). Connections are extensive among these subregions and other brain areas such as the orbitofrontal cortex, agranular insular cortex, and hippocampus. **c** Quantification analysis of the total mercury concentration in the nasal mucosa, olfactory bulb, OT, and PIR. Data are presented as the mean ± s.e.m. (*n* = 6, ^***^p < 0.001 by two-tailed Student’s *t*-test)

To investigate the effects of MeHg on the olfactory system, we established a model of subchronic MeHg toxicity (Fig. 1a). Consistent with the results of a previous study (Fujimura et al. 2009), mice exposed to MeHg for 8 weeks showed marked accumulation of mercury in the cerebral cortex and a decrease in NeuN-positive cells in the primary motor cortex (Fig. S1a, b). In addition, the mice exhibited hindlimb impairment after 5 weeks of MeHg exposure, and this impairment tended to worsen at week 8 (Fig. S1c). The mice died within 10 weeks of MeHg exposure under this dosage regimen (Fujimura et al. 2009). On the basis of these results, we determined that mice exposed to MeHg for 8 weeks had reached a sufficient level of intoxication to enable the analysis of brain disorders and other pathologies.

The main olfactory system is composed of the olfactory epithelium, which contains OSNs that receive odorants; the olfactory bulb, which is the projection site of the OSNs; and the olfactory cortex, which receives projections from the olfactory bulb (Fig. 1b). Mice exposed to MeHg for 8 weeks displayed significant mercury accumulation in the nasal mucosa, olfactory bulb, and olfactory cortex (OT and PIR) (Fig. 1c), suggesting that MeHg is broadly distributed in the olfactory pathway after exposure.

### Impairment of granule cells in the olfactory bulb by MeHg

**Fig. 2:**
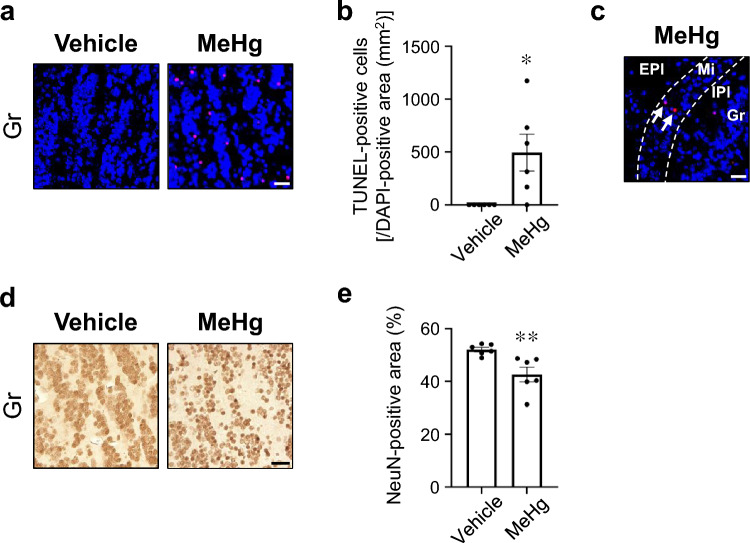
Effects of methylmercury (MeHg) exposure on the olfactory bulb. Representative images of TUNEL staining (red) in the **(a)** granule cell layer (Gr) and **(c)** mitral cell layer (Mi). Nuclei were stained with DAPI (blue). **b** Quantification of TUNEL-positive cells shown in **(a)**. The vertical axis shows the number of TUNEL-positive cells per 1 mm^2^ of DAPI-positive area. **d** Representative images of immunohistochemistry for neuronal nuclei (NeuN) in the Gr. **e** Quantification of NeuN-positive cells shown in **(d)**. The vertical axis shows the percentage of the NeuN-positive area. Data are presented as the mean ± s.e.m. (*n* = 6; ^*^p < 0.05, ^**^p < 0.01 by two-tailed Student’s *t*-test). Scale bars represent 25 μm

The olfactory bulb is a layered structure consisting of the olfactory nerve layer on the surface, followed in order by the glomerular layer, external plexiform layer (EPl), mitral cell layer (Mi), internal plexiform layer (IPl), and granule cell layer (Gr) (Schröder et al. 2020). Neuronal cell bodies are located in the glomerular layer, a shallow layer of the EPl, the Mi, and the Gr. In a previous study, apoptotic nuclei were observed in the primary motor cortex of mice after exposure to 30 ppm MeHg for 8 weeks via drinking water (Fujimura et al. 2009). To determine whether MeHg induces neuronal cell death in the olfactory bulb, we first evaluated apoptosis using the TUNEL assay. TUNEL-positive apoptotic cells significantly increased in the Gr of mice exposed to MeHg (Fig. 2a, b). The mouse with the highest mercury content in the olfactory bulb also exhibited TUNEL-positive cells in the Mi (Fig. 2c). However, such cells were not observed in any other layers except the Gr. Next, we evaluated the number of neurons by immunostaining for NeuN, a neuronal marker. Consistent with the results of the TUNEL assay, NeuN-positive neurons were significantly reduced in the Gr of mice exposed to MeHg (Fig. 2d, e). These results suggest that olfactory bulb granule cells are particularly vulnerable to MeHg exposure.

### MeHg-induced region-specific neurotoxicity in olfactory cortical areas

**Fig. 3:**
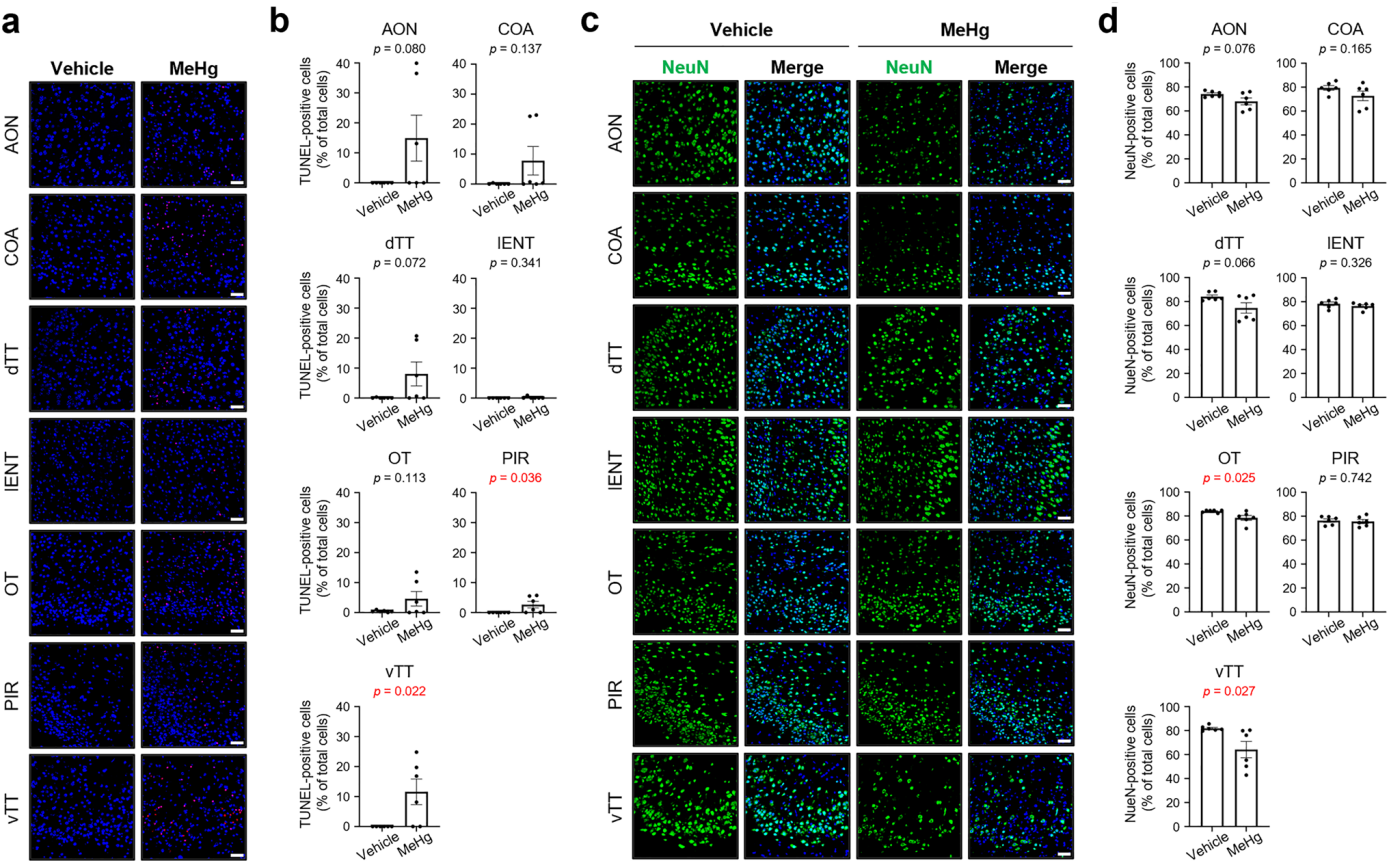
Effects of methylmercury (MeHg) exposure on the primary olfactory cortex. Representative images of **(a)** TUNEL staining (red) and **(c)** immunofluorescence for neuronal nuclei (NeuN) (green) in the primary olfactory cortex. Nuclei were stained with DAPI (blue). Quantification of **(b)** TUNEL-positive cells and **(d)** NeuN-positive cells shown in **(a)** and **(c)**. Anterior olfactory nucleus (AON), cortical amygdala (COA), dorsal tenia tecta (dTT), lateral entorhinal cortex (lENT), olfactory tubercle (OT), piriform cortex (PIR), and ventral tenia tecta (vTT). Data are presented as the mean ± s.e.m. (*n* = 6; *p*-values from two-tailed Student’s *t*-test, *p*-values in red indicate a significant difference). Scale bars represent 50 μm

Clinical studies of MD have revealed that patients show neuronal loss in the somatosensory, motor, visual, and auditory cortices (Eto and Takeuchi 1978; Takeuchi et al. 1962). However, the effects on the cortical areas involved in olfactory processing are entirely unknown. Therefore, we determined whether MeHg induces neuronal cell death in the primary olfactory cortex, which consists of the following subregions: the anterior olfactory nucleus, dorsal tenia tecta, ventral tenia tecta (vTT), OT, PIR, cortical amygdala (COA), and lateral ENT (lENT) (Schröder et al. 2020; Wilson et al. 2014). MeHg exposure increased the number of TUNEL-positive apoptotic cells in all subregions except the lENT. The increase in the number of these cells was significant in the PIR and vTT (Fig. 3a, b). In some mice exposed to MeHg, the number of NeuN-positive neurons decreased in all subregions except the PIR and lENT; however, only the OT and vTT showed significant reductions (Fig. 3c, d). These results indicate that MeHg exposure causes neuronal cell death in the olfactory cortex, particularly in the vTT. The PIR and OT also showed significant changes (Fig. 3b and d, respectively). However, the vTT, which showed significant changes in both figures, exhibited a higher rate of change than the other sites.

**Fig. 4:**
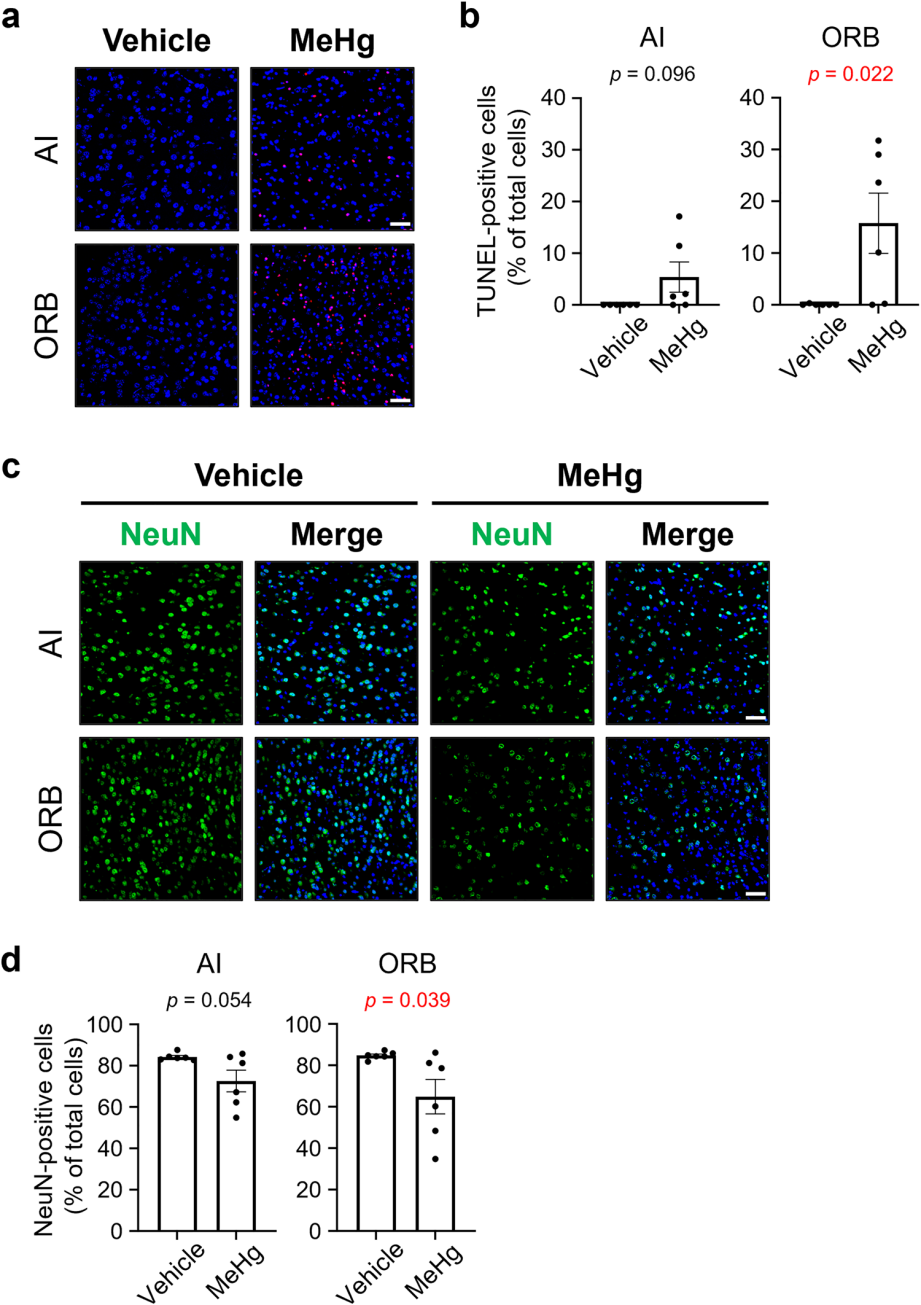
Effects of methylmercury (MeHg) exposure on the secondary olfactory cortex. Representative images of **(a)** TUNEL staining (red) and **(c)** immunofluorescence for neuronal nuclei (NeuN) (green) in the secondary olfactory cortex. Nuclei were stained with DAPI (blue). Quantification of **(b)** TUNEL-positive cells and **(d)** NeuN-positive cells shown in **(a)** and **(c)**. Agranular insular cortex (AI), orbitofrontal cortex (ORB). Data are presented as the mean ± s.e.m. (*n* = 6; *p*-values from two-tailed Student’s *t*-test, *p*-values in red indicate a significant difference). Scale bars represent 50 μm

Most subregions of the primary olfactory cortex are interconnected with higher-order secondary cortical areas such as the orbitofrontal cortex (ORB) and insula (the agranular insular cortex in rodents), which are involved in sensory integration (Mizoguchi et al. 2020; Rolls and Baylis 1994; Roy-Cote et al. 2021; Wilson et al. 2014). In the ORB, the number of TUNEL-positive apoptotic cells was significantly increased by MeHg exposure (Fig. 4a, b). Consistent with this, the number of NeuN-positive neurons was significantly reduced (Fig. 4c, d). Although not statistically significant, the agranular insular cortex showed a tendency toward a higher number of TUNEL-positive apoptotic cells and a lower number of NeuN-positive neurons in some mice exposed to MeHg (Fig. 4a–d).

### MeHg-induced activation of astrocytes and microglia in olfactory brain areas

Astrogliosis is another pathological feature of the brain in patients with MD (Eto and Takeuchi 1978; Takeuchi et al. 1962). In addition, microglial proliferation has been observed in the cerebral cortex and cerebellum of adult rodents exposed to MeHg (Fujimura et al. 2009; Sakamoto et al. 2008). Activation of these glial cells occurs near the site of neurological damage. To investigate the potential activation of astrocytes and microglia in the olfactory bulb and olfactory cortical areas, we performed immunostaining with anti-GFAP and anti-Iba1 antibodies, respectively. In the olfactory bulb of mice exposed to MeHg, GFAP-positive astrocytes became hypertrophic with greater accumulation in the EPl and Gr (Fig. S2a, b). Similar to the astrocytes, Iba1-positive microglia exhibited larger cell bodies and thicker cytoplasmic processes, taking on amoeboid forms, indicating glial activation. These activated microglia were notably more abundant in the EPl and Gr of mice exposed to MeHg (Fig. S2c, d). In the olfactory cortex, the numbers of GFAP-positive astrocytes and Iba1-positive microglia were markedly increased in all subregions except the COA and lENT after MeHg exposure (Fig. S3a–d). These results indicate that in cases of MeHg poisoning, glial activation is also a hallmark event in brain regions involved in olfactory processing.

### Correlation of neuronal loss with plasma mercury concentration

**Fig. 5:**
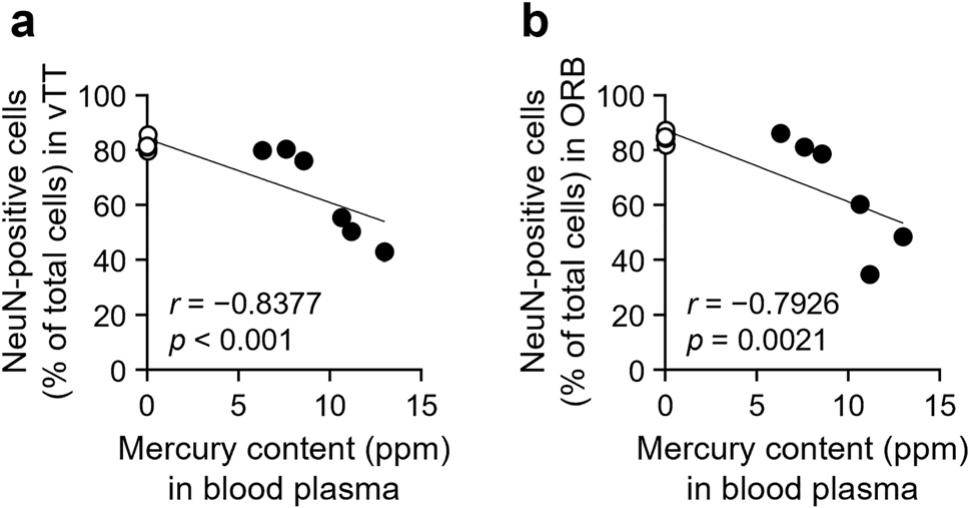
Correlation analysis between methylmercury (MeHg) exposure and neuronal loss in the olfactory cortex. Correlation between the plasma mercury concentration and the number of neuronal nuclei (NeuN)-positive cells in the **(a)** ventral tenia tecta (vTT) and **(b)** orbitofrontal cortex (ORB) after 8 weeks of exposure. Open circles, vehicle group (*n* = 6). Closed circles, MeHg-exposed group (*n* = 6). *r*, Pearson correlation coefficient

To determine whether the extent of neuronal loss in the olfactory cortex is associated with the accumulation of MeHg, we analyzed the correlation of neuronal loss with the plasma mercury concentration. The results showed a negative correlation between the number of NeuN-positive neurons in the olfactory cortex and the plasma mercury concentration (Table S1). The correlation was robust in subregions with prominent neuronal cell death, such as the vTT and ORB. Scatter plots showed that mice with plasma mercury concentrations exceeding 10 ppm had more remarkable neuronal loss (Fig. 5a, b).

### Partial disruption of OSNs by MeHg

**Fig. 6:**
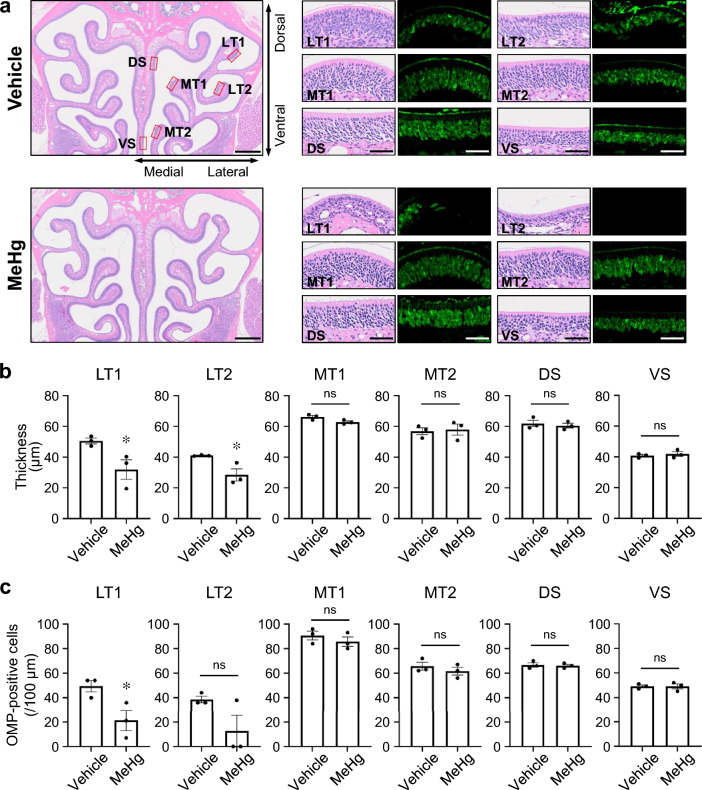
Effects of methylmercury (MeHg) exposure on the olfactory epithelium. **a** Representative images of hematoxylin and eosin staining and immunofluorescence for olfactory marker protein (OMP) (green) in the olfactory epithelium. The boxes indicate the areas of the lateral turbinate (LT1 and LT2), medial turbinate (MT1 and MT2), and dorsal/ventral nasal septum (DS and VS). Scale bars represent 500 μm (left) and 50 µm (high-magnification images). OMP signals were not detectable in LT2 of the MeHg sample. **b** Thickness of the olfactory epithelium in the six subdivisions shown in **(a)**. **c** Quantification of OMP-positive cells in the six subdivisions shown in **(a)**. The vertical axis shows the number of OMP-positive cells per 100-µm length of olfactory epithelium. Data are presented as the mean ± s.e.m. (*n* = 3; ^*^p < 0.05 by two-tailed Student’s *t*-test; ns, not significant)

Intranasal exposure to certain heavy metals is associated with damage to the olfactory epithelium (Bondier et al. 2008; Jia et al. 2010; McBride et al. 2003). Based on the data showing that mercury accumulated in the nasal mucosa after MeHg exposure to the same levels as in the cerebral cortex with neuronal loss (Figs. 1c and S1a), we predicted that the OSNs would also be damaged by MeHg. Hematoxylin and eosin staining revealed no nasal obstruction due to nasal discharge or eosinophil infiltration (Fig. 6a). To evaluate atrophy of the olfactory epithelium, the epithelial thickness was measured in six subdivisions of the bilateral nose. No differences were observed in the nasal septum and medial turbinate; however, the olfactory epithelium in the lateral turbinate was atrophic secondary to MeHg exposure (Fig. 6a, b). Next, we examined the effect of MeHg on OSNs by immunofluorescence for OMP, a marker of mature OSNs. Mice exposed to MeHg exhibited a remarkable reduction in the number of OMP-positive cells in the analyzed regions of the lateral turbinate (Fig. 6a, c; Fig. S4). These results indicate that MeHg exposure damaged the OSNs in the lateral part of the olfactory epithelium.

## Discussion

Occupational exposure to several chemicals is correlated with olfactory dysfunction (Werner and Nies 2018). Harmful chemicals are especially abundant in industrial and mineral mining areas. We easily inhaled these chemicals in the soil and air. Most animal studies investigating the olfactory toxicity of environmental chemicals have assumed inhalation exposure and evaluated toxicity to the olfactory epithelium, the pathway of inhaled air, via intranasal administration of chemicals. In contrast, MeHg is ingested orally, mainly through fish diet, and can be distributed throughout the body via the circulatory system. In addition, MeHg easily crosses the blood–brain barrier and accumulates in the brain. Although MeHg is an environmental chemical, its exposure pathways and damaged organs differ from those of other volatile and atmospherically dispersed chemicals. Therefore, the primary interest of this study was to determine how MeHg, which is neither volatile nor diffusible to the atmosphere, affects the olfactory system. In the present study, we established a model of MeHg poisoning in drinking water and found that mercury accumulates broadly in the olfactory system (Fig. 1). The granule cell layer of the olfactory bulb is particularly vulnerable to MeHg exposure (Fig. 2), and neuronal cell death occurs in cortical areas involved in olfactory processing (Figs. 3 and 4). These data indicate that MeHg has unparalleled toxicity to the olfactory system, according to its chemical properties for brain distribution. Moreover, mercury accumulated in the nasal mucosa of MeHg-intoxicated mice and the olfactory epithelium was partially atrophied (Fig. 6). This study suggests that oral exposure to environmental chemicals may also impair olfaction and may serve as a model for the toxicity analysis of chemicals with properties similar to MeHg.

Because of its affinity to thiol groups, MeHg conjugates with sulfhydryl-containing molecules such as hemoglobin, albumin, L-cysteine, and glutathione in vivo, increasing its water solubility and facilitating its distribution to various organs (Novo et al. 2021). In the present study, MeHg poisoning was induced in mice by allowing them to drink water containing MeHg–glutathione conjugates ad libitum, as previously described (Fujimura et al. 2009). Mice exposed to MeHg accumulated mercury in the cerebral cortex, as in previous analyses, and similar levels of mercury accumulation were found in the nasal mucosa and olfactory bulb (Fig. 1c and S1a). A clinical study measured the Hg concentrations in the brains of patients with acute and subacute MD (Takeuchi et al. 1962). Some patients underwent measurements relatively early after the diagnosis of MD, which exceeded 20 ppm Hg concentrations in their brains. Hg concentrations in the brains of mice exposed to MeHg shown in Fig. S1a did not deviate significantly from the values in those patients.

To determine whether MeHg accumulates in the olfactory bulb via the circulatory system, MeHg chloride (10 mg/kg body weight) was administered intraperitoneally, and increased mercury was indeed found in the nasal mucosa and olfactory bulb (Fig. S5). These data suggest that MeHg is distributed to the olfactory pathway through the circulatory system. However, we cannot completely rule out the possibility that MeHg is directly transported from the olfactory epithelium to the olfactory bulb after drinking water enters the nasal cavity. Indeed, some metals are transported to the olfactory bulb after intranasal administration, including cadmium and manganese (Tjälve et al. 1996).

Mice exposed to MeHg exhibited apoptotic cells in the Gr and Mi (Fig. 2a–c). MeHg exposure induces endoplasmic reticulum (ER) stress, C/EBP homologous protein (CHOP) expression, and apoptosis in the cerebral cortex of mice (Hiraoka et al. 2021; Nomura et al. 2022), suggesting that the Gr and Mi might be sensitive to ER stress. Intriguingly, intrabulbar infusion with tunicamycin, an N-glycosylation inhibitor and ER stress inducer, into rat pups upregulated the expression of CHOP, an ER stress-mediated pro-apoptotic transcription factor, in the Mi and Gr (Tong et al. 2017). Granule cells are γ-aminobutyric acid-releasing inhibitory interneurons that form reciprocal dendrodendritic synapses with excitatory projection neurons, including mitral cells (Isaacson and Strowbridge 1998; Yokoi et al. 1995). Granule cells appear to modulate the firing of mitral cells via inhibitory synaptic actions, enhancing olfactory discrimination between similar odors or odor mixtures (Abraham et al. 2010; Nunes and Kuner 2015; Nusser et al. 2001). Thus, MeHg-induced loss of granule cells may cause dysregulation of neuronal firing, resulting in defects in odor identification.

MD causes central sensory disturbances in visual and auditory perception (Eto 1997; Harada 1995). However, the pathophysiology of the cortical areas involved in the sense of smell remains unclear. In the present study, neuronal cell death occurred in the olfactory cortex of MeHg-exposed mice (Figs. 3 and 4). The vTT and ORB were notably vulnerable to MeHg, and the extent of neuronal loss strongly reflected the plasma mercury concentration (Fig. 5). Although the specific factors that caused the differences in the extent of neuronal loss in each subregion of the olfactory cortex remains unclear, our findings suggest that damage to the central nervous system contributes to the impaired sense of smell in patients with MD.

Several studies have shown that MeHg activates astrocytes and microglia in vivo (Ishihara et al. 2019; Shinozaki et al. 2019). Consistent with these findings, mice exposed to MeHg in the present study exhibited activated astrocytes and microglia in the olfactory bulb and olfactory cortex (Figs. S2 and S3). MeHg-induced astrocyte activation is likely neuroprotective (De Simone et al. 2017; Ishihara et al. 2019; Noguchi et al. 2013), but microglial activation may contribute to neuronal cell death (Hoshi et al. 2019; Shinozaki et al. 2019; Toyama et al. 2021). We previously reported that the number of glial cells was increased in the somatosensory cortex of mice exposed to MeHg before neuronal loss (Hiraoka et al. 2021). Because glial activation is not only a marker of MeHg-induced neuronal cell death but may also affect neuronal function, such as synaptic transmission during the early to mid-exposure period, further studies are required to elucidate the pathophysiological events that occur before the onset of neuronal cell death.

Certain heavy metals are known to injure the olfactory epithelium (Sunderman 2001); however, many elements are not toxic. Thus, the effects of heavy metals on olfaction are complex. In the present study, the lateral part of the olfactory epithelium was selectively damaged in mice exposed to MeHg (Figs. 6 and S4), suggesting that MeHg exhibits nasal toxicity. At least three factors contribute to lesion selectivity. The first is the differential expression patterns of antioxidant enzymes in the olfactory epithelium. The antioxidant enzyme NAD(P)H quinone dehydrogenase (NQO1) is abundant in some parts of the olfactory epithelium, especially around the dorsal nasal septum (Gussing and Bohm 2004). NQO1 is upregulated by MeHg and may protect against MeHg cytotoxicity (Alqahtani et al. 2023; Toyama et al. 2011). Therefore, differences in the expression patterns of antioxidant enzymes may determine resistance to MeHg in the olfactory epithelium. The second factor is the involvement of ER stress. The ER stress intensity, which depends on the olfactory receptor subtype, is a key factor that determines the projection pattern of OSN axons (Shayya et al. 2022). OSNs are unique in their use of an ER stress-related signaling pathway as a mechanism of sensing cellular identity. This pathway allows them to interpret physiological conditions and use this information to guide the wiring of their axons. The diversity in OSN responsiveness to ER stress may lead to differences in susceptibility to MeHg. The third factor is the entry of drinking water into the nasal cavity. In one study, when trypan blue was administered into the nasal cavity of mice, intense blue staining was observed in the lateral part of the olfactory epithelium (Hasegawa-Ishii et al. 2017). This indicates that the accessibility of the solution may be an underlying mechanism of specific damage to the lateral part of the olfactory epithelium in mice exposed to MeHg. These multiple factors may be responsible for the MeHg-induced damage to OSNs. On the other hand, damaged OSNs are generally regenerated by the proliferation and differentiation of basal cells. However, the effects of MeHg on the regenerative process of OSNs remain unclear. Therefore, further studies are required to fully characterize the effects of MeHg on OSNs.

Patients with MD present with olfactory disorders such as hyposmia and parosmia (Furuta et al. 1994). Hyposmia is a quantitative dysfunction that reduces the ability to smell and detect odors. It is also present in patients with Alzheimer’s disease and Parkinson’s disease and may be caused by central nervous system disorders (Ubeda-Bañon et al. 2020). Brain tissue from patients with Alzheimer’s disease and mouse models of Alzheimer’s disease have shown pathological changes and neuronal dysfunction in the ENT (Igarashi 2023; Khan et al. 2014; Stranahan and Mattson 2010), which has a solid positional and functional relationship with the hippocampus. In the present study, however, no pathological changes, such as neuronal loss or glial activation, occurred in the ENT of MeHg-intoxicated mice (Figs. 3 and S3). Furthermore, neurons in the hippocampus are more resistant to MeHg than those in the cortex (Fujimura and Unoki 2022; Fujimura et al. 2009). Thus, although both Alzheimer’s disease and MD cause olfactory dysfunction due to brain disorders, there seem to be clear differences between the brain lesions.

Parosmia is a qualitative dysfunction characterized by the inability to correctly identify an odor’s “natural” smell. The pathomechanism underlying parosmia is controversial, but several hypotheses have been proposed: partial loss of OSNs, dysfunction of the olfactory bulb by loss of interneurons, pathology of the central nervous system involved in olfactory integration/interpretation, and alterations in the olfactory map after injury, including abnormal axonal targeting of regenerated OSNs (Altundag 2023; Ciurleo et al. 2020). Consistent with these hypotheses, the present study revealed that MeHg at least partially destroys the olfactory epithelium (Fig. 6), injures granule cells (Fig. 2), and causes neuronal cell death in the olfactory cortex and higher-order cortical areas (Figs. 3 and 4). Thus, these multiple disorders may contribute to the etiology of parosmia in patients with MD.

In conclusion, our study was performed to elucidate the pathogenesis of olfactory dysfunction in MeHg poisoning. We found that MeHg injures not only the olfactory epithelium and olfactory bulb, which are vulnerable to exogenous chemicals, but also higher-order brain regions such as the olfactory cortex. These results provide further evidence that MeHg poisoning causes olfactory dysfunction as well as new insights into how environmental chemicals impair the olfactory system.

### Supplementary Information

Below is the link to the electronic supplementary material.

Supplementary file1 (DOCX 40808 KB)

## Data Availability

The datasets generated and/or analyzed during the current study are available from the corresponding author on reasonable request.
