# Supplementary material for: Characterization of pathological changes in the olfactory system of mice exposed to methylmercury
Source: Arch Toxicol. 2024 Feb 17;98(4):1163–75. doi: 10.1007/s00204-024-03682-w (PMC10944439; doi:10.1007/s00204-024-03682-w)
Supplement: Supplementary file 1 — Supplementary file1 (DOCX 40808 KB) [file 204_2024_3682_MOESM1_ESM.docx]

**Supplemantary Information:**

**Characterization of pathological changes in the olfactory system of mice exposed to methylmercury**

**Yuta Iijima**^1^**, Ryohei Miki**^1^**, Nobumasa Takasugi**^1^**, Masatake Fujimura**^2^**, and Takashi Uehara**^1, *^

^1^ Department of Medicinal Pharmacology, Graduate School of Medicine, Dentistry and Pharmaceutical Sciences, Okayama University, Okayama 700‑8530, Japan

^2^ Department of Basic Medical Science, National Institute for Minamata Disease, Kumamoto 867‑0008, Japan.

* Corresponding author, email: [uehara-t@okayama-u.ac.jp](mailto:uehara-t@okayama-u.ac.jp)

**
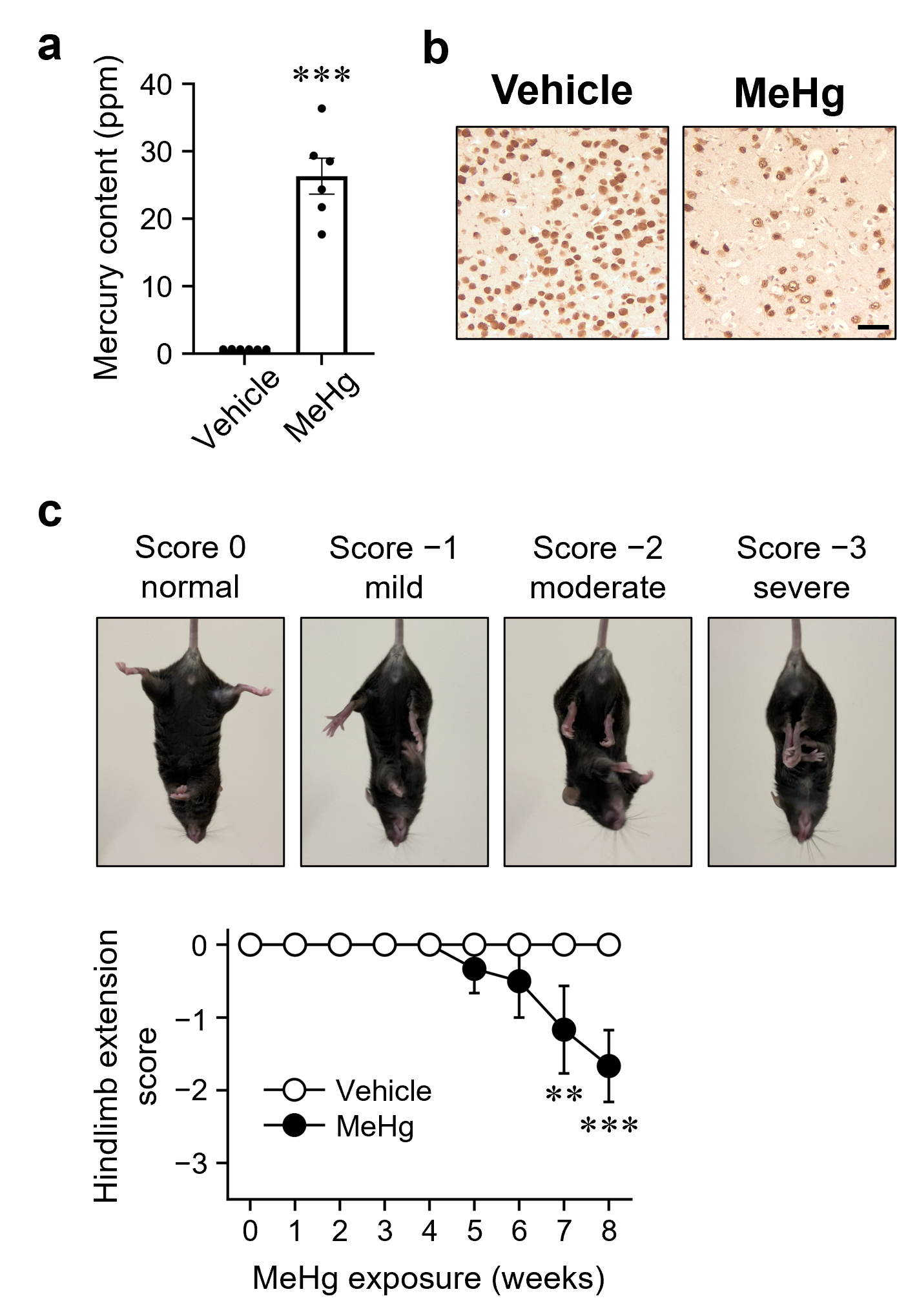
**

**Fig. S1** Mouse model of subchronic MeHg exposure. **a** Quantification analysis of total mercury concentration in the cerebral cortex of wild-type mice exposed to vehicle or MeHg for 8 weeks (*n* = 6; ^***^*p* < 0.001 by two-tailed Student’s *t*-test). **b** Representative images of immunostaining for NeuN in the primary motor cortex. The scale bar represents 50 μm. **c** Photographs of the hindlimb extension test and quantification using a −3 to 0 numerical scale; 0, normal phenotype; −1, mild defect; −2, moderate defect; −3, severe defect. Data are presented as the mean ± s.e.m. (*n* = 6; ^**^*p* < 0.01, ^***^*p* < 0.001 by two-way ANOVA followed by Bonferroni’s post hoc test).

**
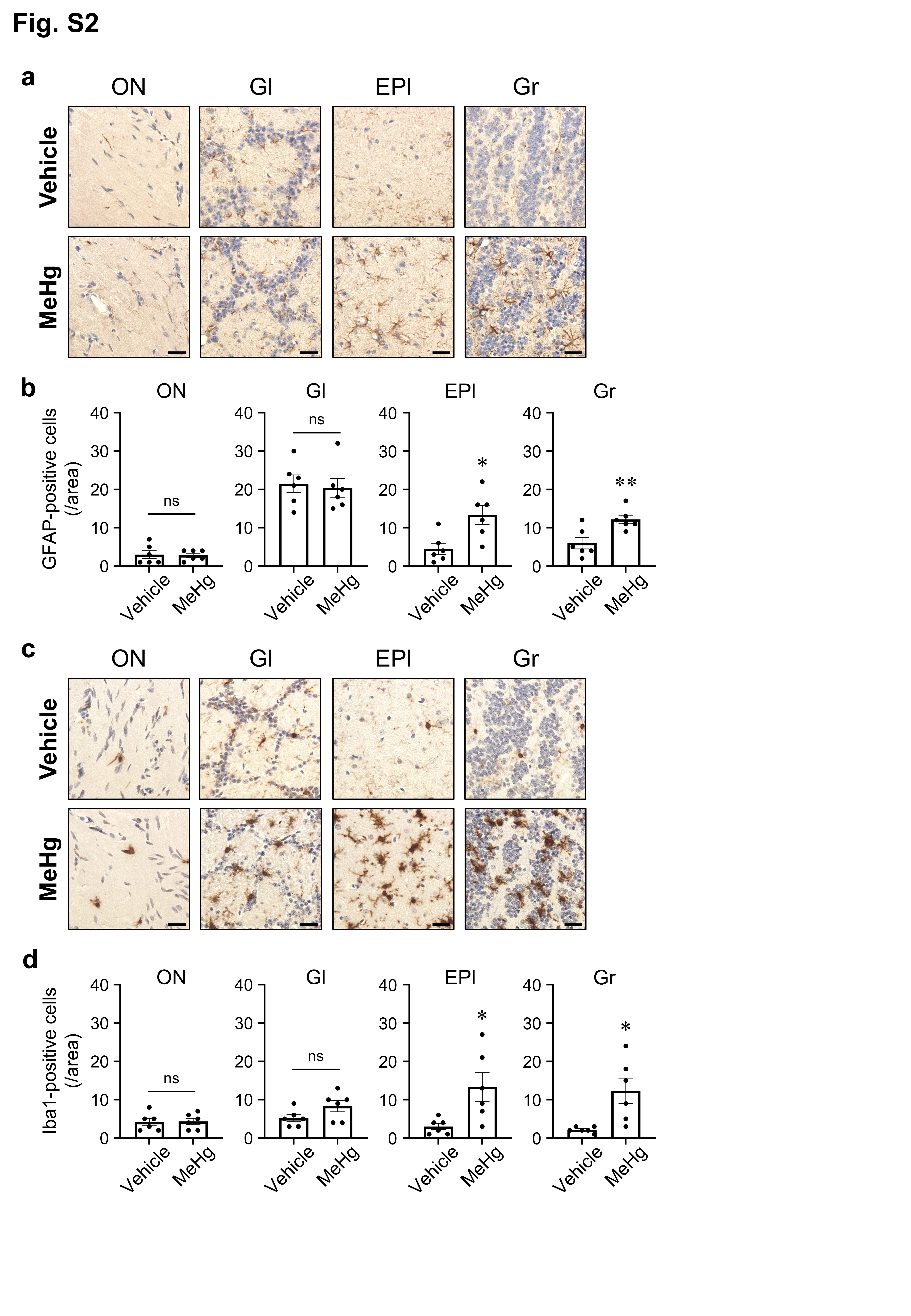
**

(Continued below)

**Fig. S2** Glial response to MeHg in the olfactory bulb. Representative images of immunostaining for **(a)** GFAP and **(c)** Iba1 in the olfactory bulb. Nuclei were stained with hematoxylin. Scale bars represent 25 µm. Quantification of **(b)** GFAP-positive cells and **(d)** Iba1-positive cells shown in **(a)** and **(c)**. The vertical axis shows the number of immunopositive cells per 169- × 169- µm^2^ area. Olfactory nerve layer (ON), glomerular layer (Gl), external plexiform layer (EPl), and granule cell layer (Gr). Data are presented as the mean ± s.e.m. (*n* = 6; ^*^*p* < 0.05, ^**^*p* < 0.01 by two-tailed Student’s *t*-test; ns, not significant).

**
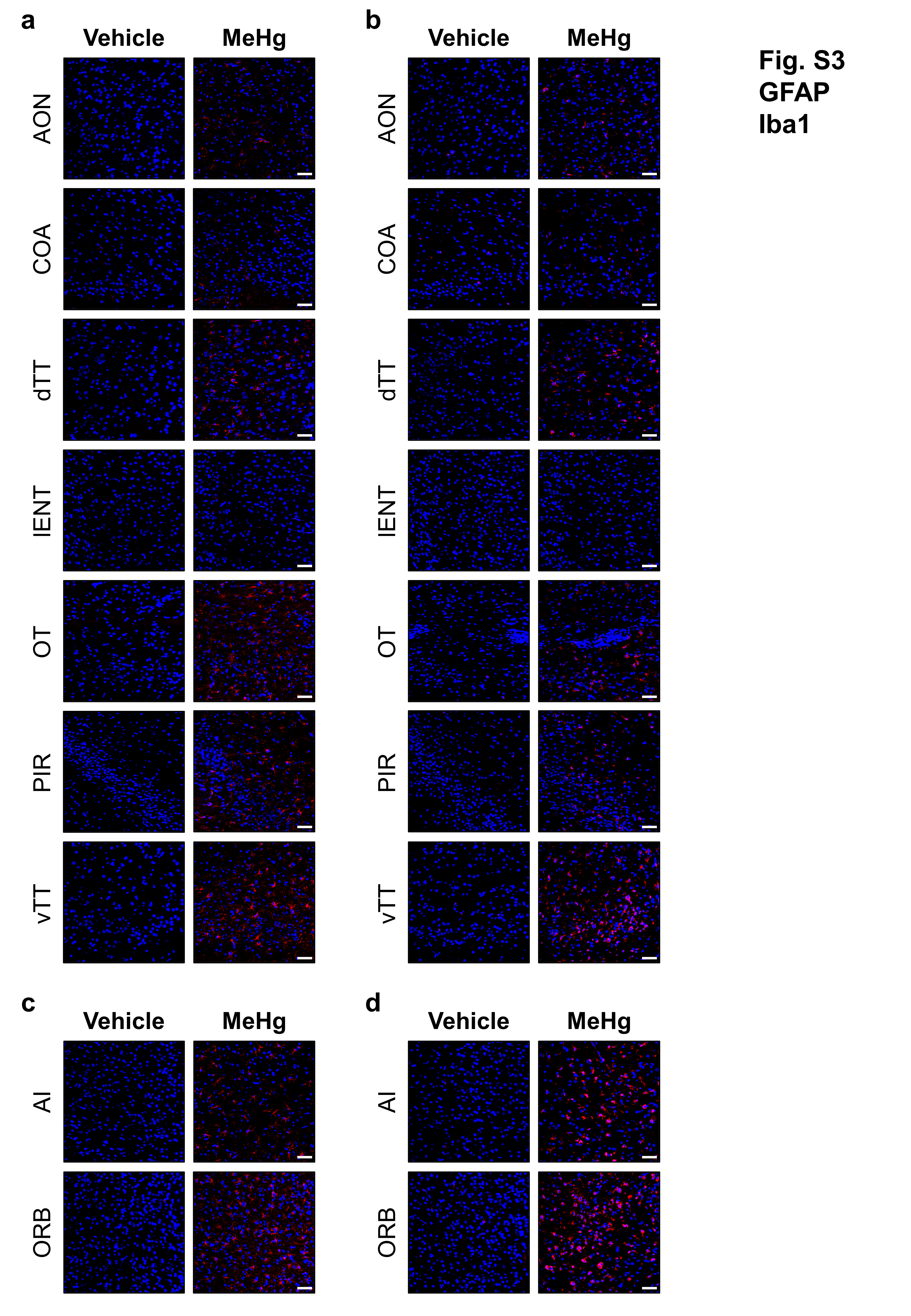
**

(Continued below)

**Fig. S3** Glial response to MeHg in the olfactory cortex. Representative images of immunofluorescence for GFAP (red) in the **(a)** primary olfactory cortex and **(c)** secondary olfactory cortex. Representative images of immunofluorescence for Iba1 (red) in the **(b)** primary olfactory cortex and **(d)** secondary olfactory cortex. Nuclei were stained with DAPI (blue). Anterior olfactory nucleus (AON), cortical amygdala (COA), dorsal tenia tecta (dTT), lateral entorhinal cortex (lENT), olfactory tubercle (OT), piriform cortex (PIR), ventral tenia tecta (vTT), agranular insular cortex (AI) and orbitofrontal cortex (ORB). Scale bars represent 50 µm.

**
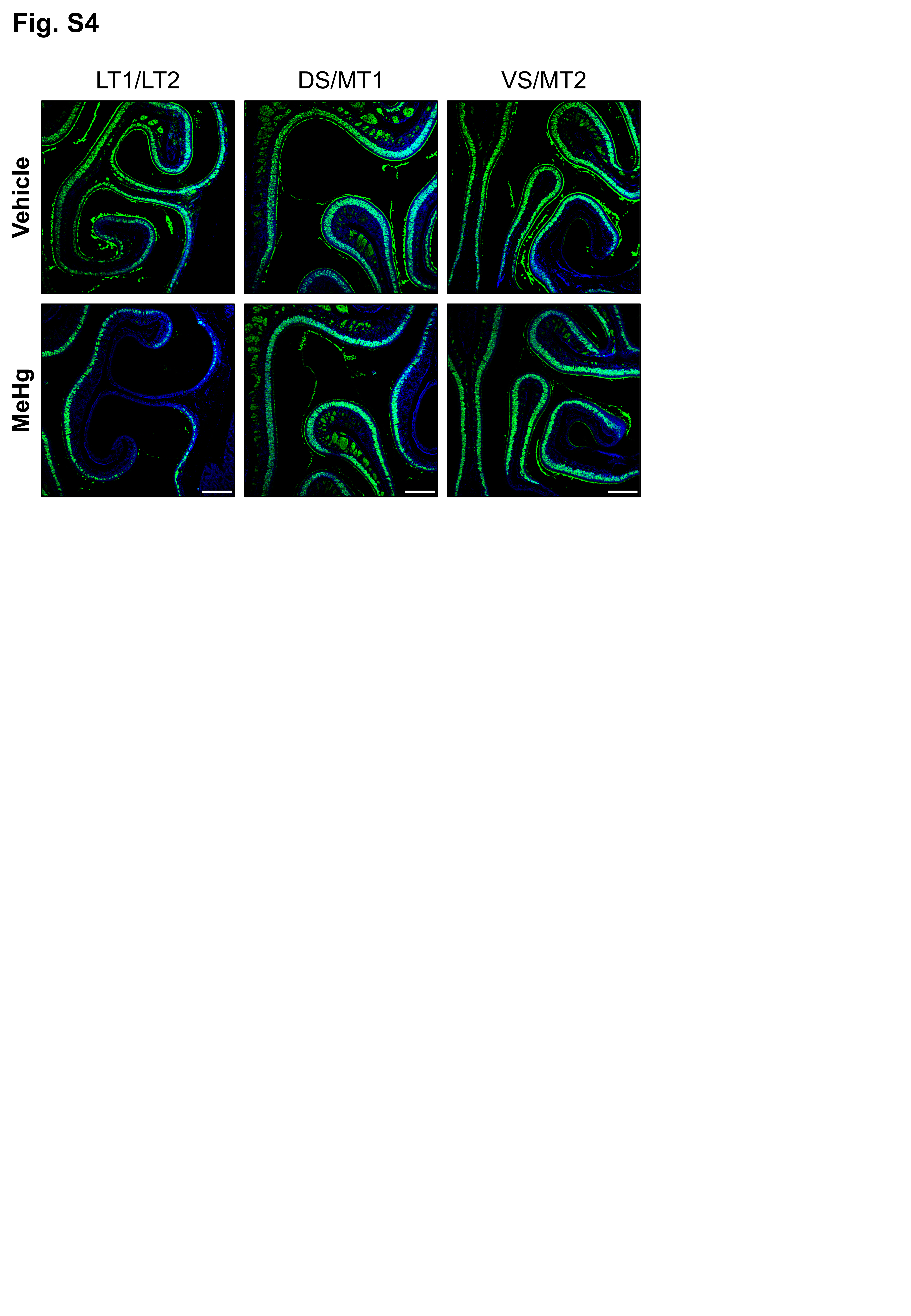
**

**Fig. S4** Loss of olfactory sensory neurons by MeHg exposure. Low-magnification images of immunofluorescence for OMP (green) shown in Fig. 6a. The images display the surrounding areas of the lateral turbinate (LT1 and LT2), medial turbinate (MT1 and MT2) and dorsal/ventral nasal septum (DS and VS). Nuclei were stained with DAPI (blue). The OMP signals were partially lost in LT1/LT2 area. Scale bars represent 200 μm.

**Table S1.** Correlation between neuronal loss in the region of interest and mercury concentration in blood plasma.

| **Region of interest** | **Pearson correlation**  **coefficient (*r*)** | ***p*-value** | ***p*-value**  **summary** |
| --- | --- | --- | --- |
| AI | −0.7727 | 0.0032 | ** |
| AON | −0.7406 | 0.0059 | ** |
| COA | −0.6131 | 0.0340 | * |
| dTT | −0.7544 | 0.0046 | ** |
| lENT | −0.3390 | 0.2811 | ns |
| OT | −0.7109 | 0.0095 | ** |
| ORB | −0.7926 | 0.0021 | ** |
| PIR | −0.09475 | 0.7696 | ns |
| vTT | −0.8377 | < 0.001 | *** |

Abbreviations: AI, agranular insular cortex; AON, anterior olfactory nucleus; COA, cortical amygdala; dTT, dorsal tenia tecta; lENT, lateral entorhinal cortex; OT, olfactory tubercle; ORB, orbitofrontal cortex; PIR, piriform cortex; vTT, ventral tenia tecta; ns, not significant. ^*^*p* < 0.05, ^**^*p* < 0.01, ^***^*p* < 0.001 by Pearson correlation.

**
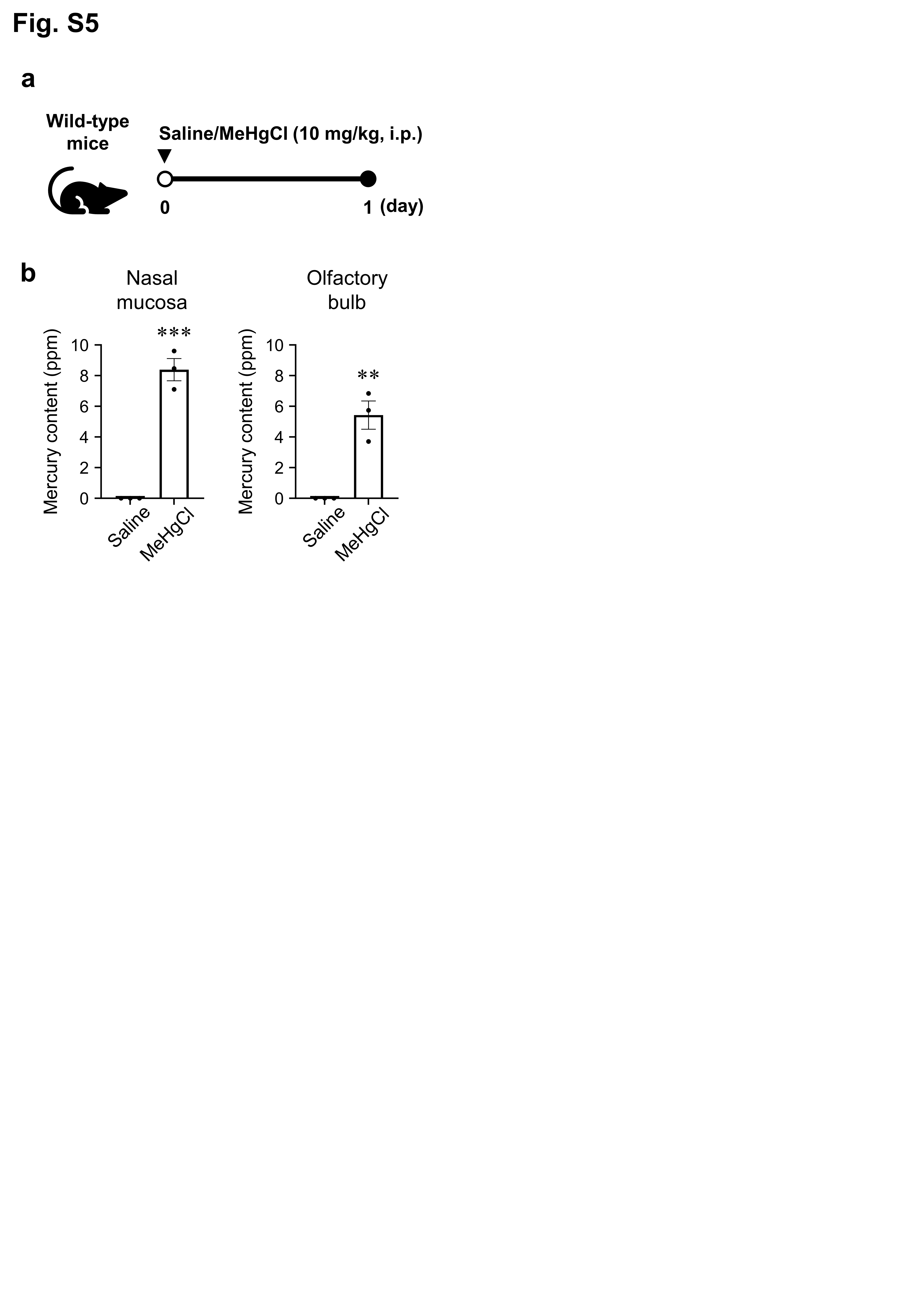
**

**Fig. S5** Distribution of mercury to the olfactory pathway. **a** Schematic of intraperitoneal injection (i.p.) of methylmercury chloride (MeHgCl) and saline. After 24 hours of injection, the mice were sacrificed for measurements of mercury content shown in **(b)**. **b** Quantification analysis of total mercury concentration in the nasal mucosa and olfactory bulb. Data are presented as the mean ± s.e.m. (*n* = 3; ^**^*p* < 0.01, ^***^*p* < 0.001 by two-tailed Student’s *t*-test).
